# HIV infection and drugs of abuse: role of acute phase proteins

**DOI:** 10.1186/1742-2094-10-113

**Published:** 2013-09-17

**Authors:** Thangavel Samikkannu, Kurapati VK Rao, Adriana Y Arias, Aarthi Kalaichezian, Vidya Sagar, Changwon Yoo, Madhavan PN Nair

**Affiliations:** 1 Department of Immunology, Institute of NeuroImmune Pharmacology, College of Medicine, Florida International University, Miami, FL 33199, USA; 2 Department of Epidemiology and Biostatistics, School of Public Health, Florida International University, Miami, FL 33199, USA

**Keywords:** Drugs of abuse, HIV, Plasma, C-reactive protein, Serum amyloid A

## Abstract

**Background:**

HIV infection and drugs of abuse such as methamphetamine (METH), cocaine, and alcohol use have been identified as risk factors for triggering inflammation. Acute phase proteins such as C-reactive protein (CRP) and serum amyloid A (SAA) are the biomarkers of inflammation. Hence, the interactive effect of drugs of abuse with acute phase proteins in HIV-positive subjects was investigated.

**Methods:**

Plasma samples were utilized from 75 subjects with METH use, cocaine use, alcohol use, and HIV-positive alone and HIV-positive METH, cocaine, and alcohol users, and age-matched control subjects. The plasma CRP and SAA levels were measured by ELISA and western blot respectively and the CD4 counts were also measured.

**Results:**

Observed results indicated that the CRP and SAA levels in HIV-positive subjects who are METH, cocaine and alcohol users were significantly higher when compared with either drugs of abuse or HIV-positive alone. The CD4 counts were also dramatically reduced in HIV-positive with drugs of abuse subjects compared with only HIV-positive subjects.

**Conclusions:**

These results suggest that, in HIV-positive subjects, drugs of abuse increase the levels of CRP and SAA, which may impact on the HIV infection and disease progression.

## Background

Inflammatory proteins such as C-reactive protein (CRP) and serum amyloid A (SAA) are the plasma proteins known as acute phase proteins that increase during systemic inflammation. These proteins are present in blood and are abundantly expressed in the liver [1,2]. CRP binds with highest affinity to phosphocholine residues and modifies plasma lipoproteins [3], which alters antioxidant defense mechanism to promote apoptosis [4]. The production of SAA mainly induces proinflammatory cytokines such as TNFα, IL-6 and IL-1. SAA seems to be involved in circulating the main fibril protein of secondary amyloidosis and is associated with an increased level of metalloproteinase, collagen synthesis, and increased extracellular matrix. An increased level of SAA binds to lipoproteins during inflammation, which is implicated in atherosclerosis and thrombosis [5]. Acute phase proteins have been associated with immune dysfunction and subsequently affect inflammatory cytokines [6], psychological and psychiatric illnesses and pathogens including HIV infection [2,7,8]. Studies have shown that Alzheimer’s disease patients with increased chronic inflammation have elevated levels of CRP and SAA in the brain [9,10]. In dementia patients, CRP and SAA participate in diverse cellular functions including stress, depression and cognitive neuronal disorders [11,12]. The acute phase proteins CRP and SAA play a central role in the molecular mechanisms triggered after immune dysfunction in the cardiovascular complication and neuropathogenesis of HIV disease progression [13,14].

Alcohols as well as illicit drugs such as methamphetamine (METH) and cocaine are powerful psychostimulants that are widely abused in the USA. Illicit drug abuse is a risk factor that plays a significant role in HIV infection and AIDS disease progression. According to the National Household Survey on Drug Abuse, in 2010 about 22.6 million Americans (aged 12 or older) were illicit drug abusers. Approximately 4.8 million Americans age 12 and older had abused cocaine in any form and 1.0 million had abused crack/cocaine at least once in a year. In alcohol users almost one-half of Americans aged 12 or older (51.8%) reported being current drinkers, translating to an estimated 131.3 million people. In 2010, a revised report from the National Survey on Drug Use and Health estimated 13 million people age 12 or older (4.3% of the population) have tried METH [15,16]. Studies have shown that illicit drugs are risk factors for triggering inflammation and immune functions [17,18]. These drugs also play an important role in the etiology of viral infection and disease progression. Previous studies suggested that drug abuse, crack/cocaine, METH and alcohol are risk factors for contracting HIV-1 infection and have been shown to be independently associated with progression to clinical AIDS [19-21]. Studies have reported that increased inflammatory proteins CRP and SAA in HIV-infected subjects [22,23] contribute to immune suppression and disease progression [24]. Alcohol users and abusers of METH and cocaine develop chronic inflammation that leads to immune dysfunction as well as neuronal impairments. The interactive role for HIV progression and exacerbation of acute phase proteins CRP and SAA linkage remains to be studied. The aim of the present study was to investigate the levels of CRP and SAA in METH, cocaine and alcohol users and the effects on their interactive role with HIV infection and disease progression.

## Methods

### Patient population

Human subjects were recruited from collaborating physicians and community agencies, as well as by participant referral. After eligibility was confirmed, informed consent was obtained. Participants provided medical documentation to confirm HIV and hepatitis B and C status. For HIV-positive individuals, medical documentation confirmed CD4 and viral loads. Individuals younger than 18 years old and with hepatitis B and/or hepatitis C infection were deemed ineligible. Self-report data were collected concerning drug use history, and participant’s blood specimens were collected by a registered nurse. All researchers coming into contact with human subjects had obtained the required collaborative Institutional Review Board training initiative. The identity of participants was concealed to maintain participant confidentiality.

### Immunoassay of C-reactive protein and serum amyloid A in plasma

Blood was collected from HIV-infected patients and substance abusers 24 to 72 hours after the last use of METH, cocaine, alcohol and marijuana. The blood samples were centrifuged after collection and residual plasma specimens were stored at −80°C. CRP was measured in plasma by a commercial immunoassay kit (Immunoassay Kit #MBS 564038; MBiosource, San Diego, CA, USA). The commercial kit uses a mAb raised against full-length human CRP to detect and quantify the level of total protein. The SAA was measured in plasma by another commercial immunoassay kit (Immunoassay Kit # ST1056; Calbiochem, San Diego, CA, USA). This commercial kit uses a mouse mAb raised against full-length human SAA to detect and quantify the level of total protein.

### Western blot analysis of plasma C-reactive protein and serum amyloid A

To assess the randomly selected plasma CRP and SAA protein levels from either METH, cocaine, and alcohol users, HIV-positive subjects alone or HIV-positive with METH, cocaine and alcohol users, equal amounts of plasma proteins were resolved on a 4 to 15% gradient SDS-PAGE, transferred to a nitrocellulose membrane and incubated with CRP antibodies (Biolegent, San Diego, CA, USA) and SAA antibodies (Calbiochem) and their respective secondary antibodies. Immunoreactive bands were visualized using a chemiluminescence western blotting system according to the manufacturers’ instructions (Amersham).

### Data analysis

Statistical calculations were carried out using GraphPad Prism 5 Statistical Software Package (GraphPad Software Inc., La Jolla, CA, USA). The interactive effect of either METH, cocaine or alcohol user with HIV-positive subjects compared with control subjects were calculated using analysis of variance and the Student’s *t* test as well as the nonparametric Mann–Whitney U test. Significance of comparisons between normal subjects and subjects with either cocaine or alcohol use with HIV-positive subjects is denoted by *P* <0.05.

## Results

Plasma samples from alcohol, METH, and cocaine users and HIV-positive subjects alone or HIV-positive with METH, cocaine and alcohol users were compared with normal subjects. The normal drug-free groups consisted of eight subjects and were healthy, normally developing subjects, unrelated to the diseased patients.

**Figure 1:**
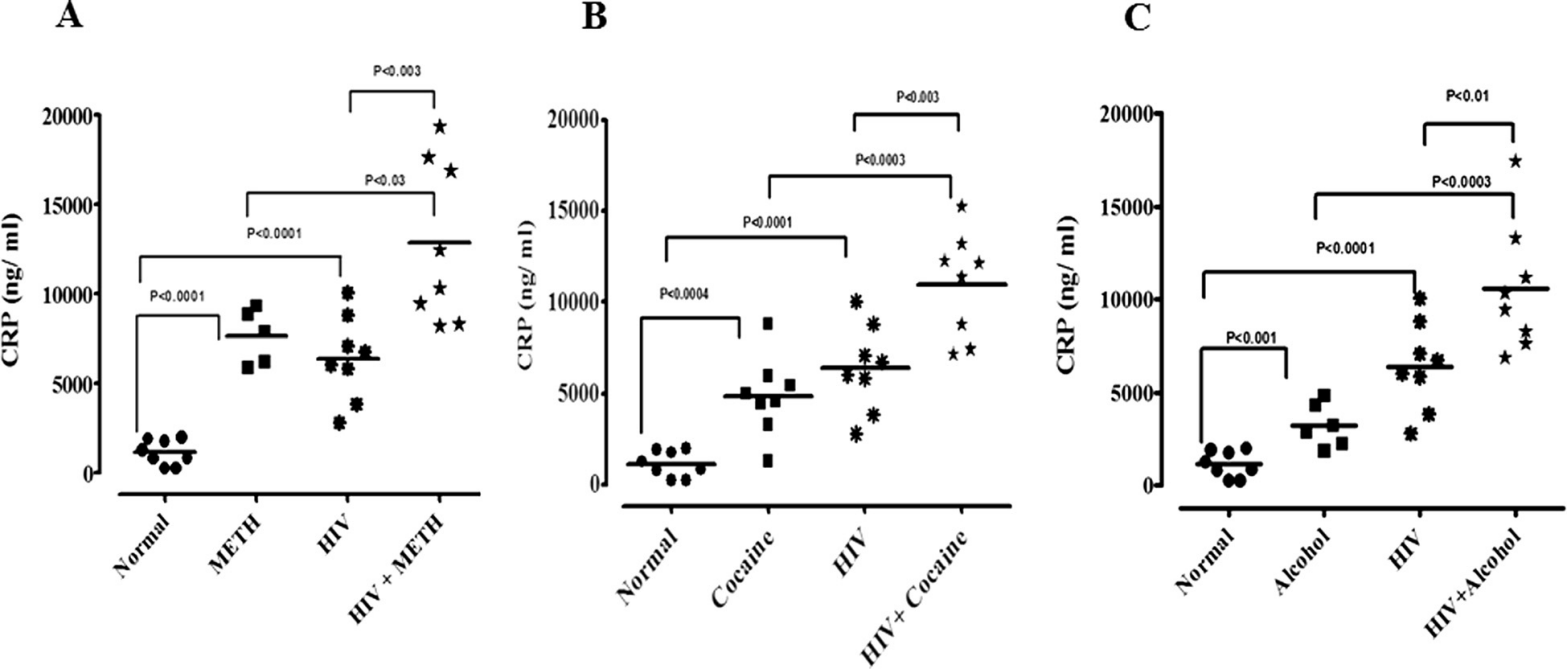
**Plasma levels of C-reactive protein estimated in HIV-positive drug users.** Plasma levels of C-reactive protein (CRP) estimated by ELISA in **(A)** methamphetamine (METH), **(B)** cocaine and **(C)** alcohol users with HIV-positive. Horizontal lines indicate the mean ± standard error of the mean.

**Figure 2:**
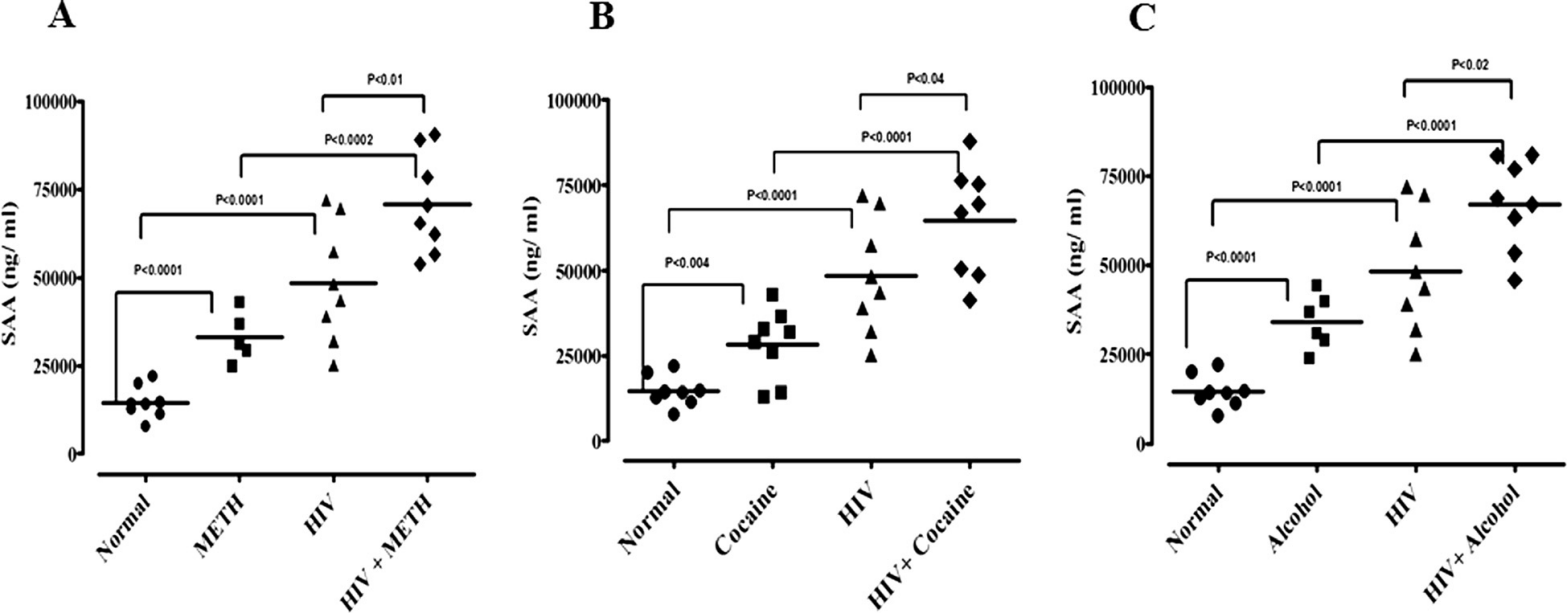
**Plasma levels of serum amyloid A protein estimated in HIV-positive drug users.** Plasma levels of serum amyloid A (SAA) protein estimated by ELISA in **(A)** methamphetamine (METH), **(B)** cocaine and **(C)** alcohol users with HIV-positive. Horizontal lines indicate mean ± standard error of the mean.

Plasma was analyzed for CRP and SAA by ELISA in METH, cocaine, and alcohol users and HIV-positive alone or in drugs of abuse METH, cocaine, and alcohol with HIV-positive subjects. Figure 1 shows the significantly elevated levels of plasma CRP in HIV-positive with METH, cocaine and alcohol users compared with just METH, cocaine and alcohol users, and HIV-positive subjects. Similarly, Figure 2 shows significantly elevated levels of plasma SAA in HIV-positive with METH, cocaine and alcohol users compared with just METH, cocaine and alcohol users and HIV-positive subjects alone. The plasma CRP and SAA levels in METH, cocaine and alcohol users and HIV-positive subjects when compared with normal subjects significantly increased, whereas these effects were significantly less when compared with combination of HIV-positive with METH, cocaine and alcohol users.

**Figure 3:**
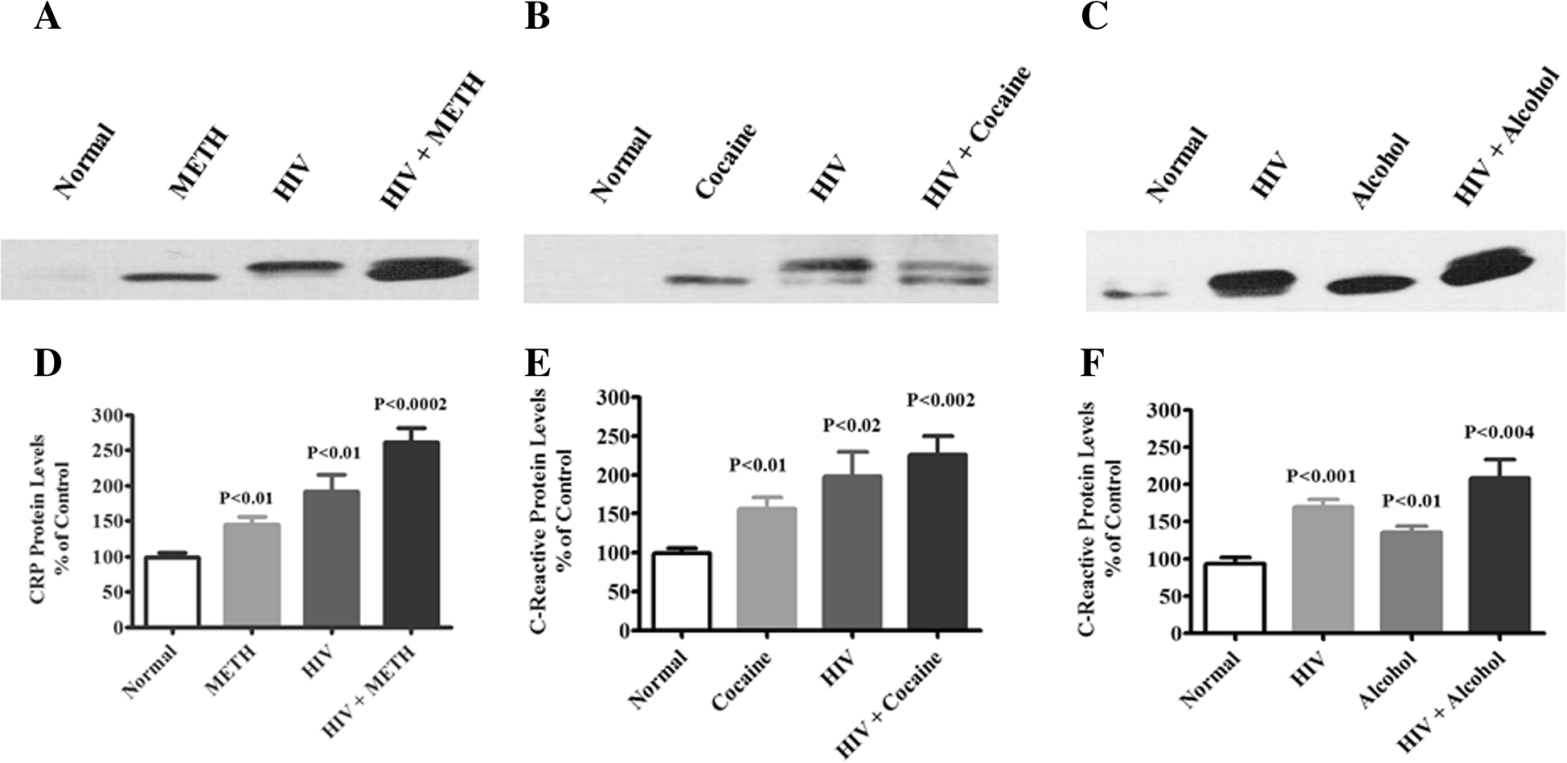
**Plasma C-reactive protein expression in HIV-positive drug users.** Plasma C-reactive protein (CRP) expression elucidated by western blots in **(A)** HIV-positive methamphetamine (METH) users, **(B)** HIV-positive cocaine users and **(C)** HIV-positive alcohol users compared with age-matched normal subjects. **(D)**, **(E)**, **(F)** Represented percentage densitometric values of CRP protein levels (% control). Data expressed as mean ± standard error of the mean of randomly selected in each groups for three subjects.

**Figure 4:**
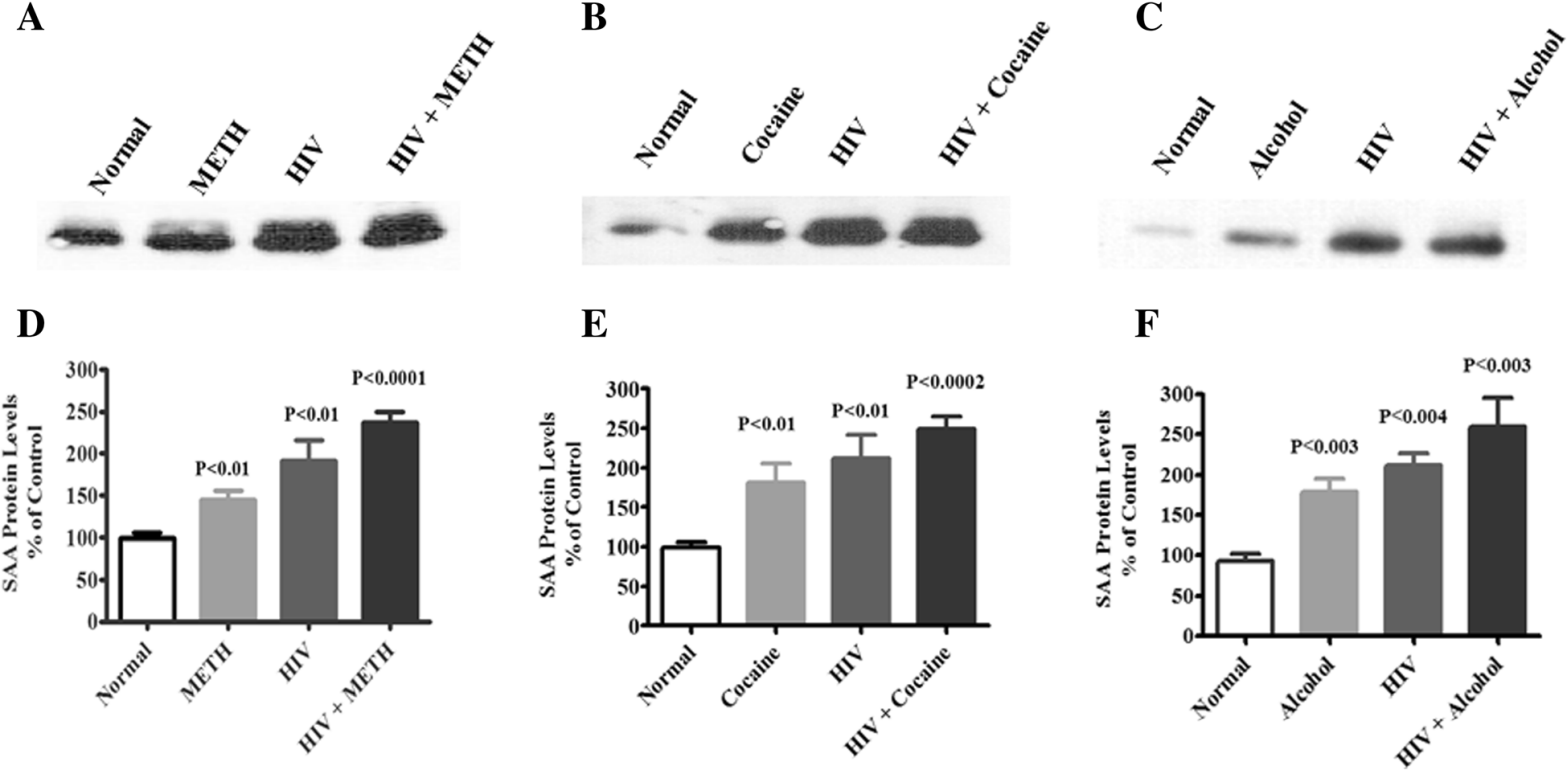
**Plasma serum amyloid A protein expression in HIV-positive drug users.** Plasma serum amyloid A (SAA) protein expression elucidated by western blots in **(A)** HIV-positive methamphetamine (METH) users, **(B)** HIV-positive cocaine users and **(C)** HIV-positive alcohol users compared with age-matched normal subjects. **(D)**, **(E)**, **(F)** Represented percentage densitometric values of SAA protein levels (% control). Data expressed as mean ± standard error of the mean of randomly selected in each groups for three subjects.

To validate the increased levels by ELISA, we also investigated plasma levels of CRP and SAA protein by western blot analysis in randomly selected plasma samples from normal subjects, METH, cocaine, and alcohol users, HIV-positive subjects alone and cocaine, METH and alcohol users with HIV-positive subjects. The protein expression of CRP and SAA is represented in Figures 3 and 4 respectively in HIV-positive subjects with METH abuse, HIV-positive subjects with cocaine abuse and HIV-positive subjects with alcohol use compared with controls. Figures 3 and 4D,E,F represent densitometric evaluations of the statistically significant difference respectively in METH, cocaine and alcohol users with HIV-infected subjects. A concordance was observed between the elevated levels of CRP and SAA by ELISA and western blotting analysis. The observed results of acute phase proteins CRP and SAA inversely correlated with CD4 counts and were significantly reduced in METH, cocaine, and alcohol users with HIV-infected subjects compared with HIV-infected subjects (Table 1).

**Table 1:** Baseline characteristics of HIV-positive subjects with methamphetamine, cocaine and alcohol users

	**Normal subjects**	**HIV-positive subjects**	**HIV-positive**		
			**Methamphetamine users**	**Cocaine users**	**Alcohol users**
**Age**	**39.23 ± 2.32**	**48.38 ± 1.75**	**45.27 ± 2.08**	**47.75 ± 1.30**	**43.75 ± 2.63**
**Sex**	**Male 6**	**Male 5**	**Male 6**	**Male 5**	**Male 6**
	**Female 2**	**Female 3**	**Female 2**	**Female 3**	**Female 2**
**CD4 cell**	**–**	**≥406/μl**	**≥186/μl**	**≥214/μl**	**≥237/μl**

## Discussion

Studies have reported that HIV infections are enhanced by METH, cocaine and alcohol use *in vitro* and *in vivo*[21,25,26]. Population and clinical studies have demonstrated that illicit drugs interact with HIV-positive subjects and accelerate viral replication and disease progression synergistically, by impairing immune and neuronal functions [18,27,28]. We measured acute phase proteins CRP and SAA in plasma from METH, cocaine and alcohol users alone or from HIV-positive subjects with drugs of abuse cocaine, METH and alcohol subjects using the ELISA method. The expressions of both acute phase protein CRP and SAA levels were significantly elevated when compared with age-matched control groups. Furthermore, there was a significant association between CRP and SAA levels in METH, cocaine and alcohol users with HIV infection associated with reduced CD4 counts (Table 1). The plasma levels of acute phase proteins were positively correlated with METH, cocaine and alcohol users alone and HIV-positive subjects, providing additional evidence for increased disease progression and immune susceptibility level of inflammatory markers CRP and SAA.

Studies have shown that CRP and SAA level are elevated in cocaine abusers, alcohol users and cigarette smokers [19,20,29]. The inflammatory markers CRP and SAA proteins play a major role in chronic inflammation of HIV infection, inducing immune dysfunction, disease progression and subsequently decreased CD4 count [22]. Recent work demonstrated that the level of inflammatory marker CRP was elevated in patients with intracerebral hemorrhage in alcohol users [30]. Cocaine abusers had fivefold to sixfold increases in the levels of CRP, which lead to sudden heart attack and may also trigger fatal cardiovascular events such as strokes [19]. In addition, chronic inflammatory changes that occur during stress and depression subsequently lead to dementia. CRP plays a wide role in the development of cardiovascular complications, dementia, cognitive impairments and Alzheimer’s disease. Recent studies report that HIV-positive patients showed increased levels of CRP compared with the HIV-negative population [22], although CRP and SAA levels in METH, cocaine and alcohol users were not compared with HIV-infected subjects. Previous research has reported that the inflammatory acute phase proteins CRP and SAA were increased in cocaine and alcohol users [19,20]. These studies are consistent and suggest that at least part of the immunopathogenic mechanisms related to METH, cocaine and alcohol users may be due to a persistent state of subclinical inflammation. We observed that an elevated expression of CRP and SAA in METH, cocaine and alcohol subjects is associated with the HIV-positive subjects. Cocaine, and alcohol are frequently used together [31] and this combination is associated with enhanced immune and central nervous system toxicity and dependence severity [32]. These results suggest that the levels of acute phase proteins CRP and SAA may serve as biomarkers of illicit drug users and HIV-infected subjects.

These inflammatory proteins CRP and SAA are the most dramatic acute phase reactants, and are associated with high-density lipoproteins. Normally SAA levels are present at <5 μg/ml in plasma; however, during activation the protein levels increase more than 500-fold by acute phase and subsequently lead to psychomotor dysfunction. However, studies have reported that the apolipoprotein E4 allele has been a major player in dementia and peripheral neuropathy [33]. Also, studies have shown controversial reports in apolipoprotein E4 dependence and independence in HIV-associated dementia [33,34]. This specific allele of the apolipoprotein E4 region has been associated with increasing oxidative stress and is very sensitive in HIV-associated dementia patients. However, studies have supported that CRP acts as an indicator of bacterial and viral infection and it is linked with the apolipoprotein E4 allele. The higher level of CRP associated with apolipoprotein E4 has been reported to exacerbate the susceptibility of dementia and cognitive impairments [35]. Studies have reported that reduced plasma tryptophan increased neuronal dysfunction including behavioral and cardiovascular impairments [36,37]. However, HIV infection and disease progression are majorly activated by tryptophan catabolism in HIV-infected subjects, illicit drug use, as well as alcohol users [38,39], and further tryptophan deficiencies targeting CD4 cells. The altered tryptophan catabolism changes to acidic pH in blood plasma, resulting in negative charge in CRP [40]. This result suggests that acute phase proteins lead tryptophan deficiencies in drugs of abuse and HIV-infected subjects and may play an interactive role with acceleration of viral replication and disease progression.

## Conclusions

In this study we examined the interactive role of illicit drugs in HIV-positive subject’s plasma proteins CRP and SAA in order to understand their role in HIV pathogenesis. Our results clearly indicate that drugs of abuse such as METH, cocaine and alcohol are capable of accelerating the secretion of CRP and SAA levels and subsequently decrease the CD4 cell count, which causes chronic inflammatory effects. These observations lead to the hypothesis that acute phase proteins CRP and SAA play a major role and contribute to viral replication and disease progression in drug abusers.

## Abbreviations

CRP: C-reactive protein; ELISA: Enzyme-linked immunosorbent assay; IL: Interleukin; mAb: Monoclonal antibody; METH: Methamphetamine; SAA: Serum amyloid A; TNF: Tumor necrosis factor.

## Competing interests

The authors declare that they have no competing interests.

## Authors’ contributions

All authors read and approved the final manuscript.
